# Coumestrol induces apoptosis and inhibits invasion in human liver cancer cells

**DOI:** 10.1016/j.toxrep.2025.102091

**Published:** 2025-07-19

**Authors:** Hoda Abdul-Nabie, Safia Samir, Tarek Aboshousha, Nagui Fares, Faten Sabra Abo-Zeid

**Affiliations:** a Zoology Department, Faculty of Science, Ain Shams University, Egypt; b Biochemistry and Molecular Biology Department, Theodor Bilharz Research Institute, Giza, Egypt; c Pathology Department, Theodor Bilharz Research Institute, Giza, Egypt

**Keywords:** Expression of genes, Quantitative real-time PCR, Liver cancer, Coumestrol, Apoptosis, Immunohistochemistry

## Abstract

Hepatocellular carcinoma (HCC) is a major public health problem, with a poor prognosis in patients with advanced disease. We aimed to investigate the cytotoxic activity of coumestrol against human hepatocellular carcinoma HepG2 cells. Crystal violet (CV) assay was performed for cell cytotoxicity assessment on Vero cells (normal Kidney) and HepG2 cells. Apoptosis was analyzed by Annexin V/FITC staining. The relative expression of BAX, bcl-2, NF-Kβ, PCNA, MMP2, Caspase-3, Caspase-9, COX2, and MMP9 was detected using quantitative real-time PCR (qPCR). In addition, immunohistochemical analysis (IHC) of Bax, and PCNA were established. Results indicated that coumestrol induced significant toxicity in HepG2 cells. Annexin V-FITC staining assays revealed that coumestrol-induced cytotoxicity in HepG2 cells was mediated through apoptosis stimulation. The apoptosis in HepG2 cells was mediated through caspase-activation. Cell invasion was inhibited by coumestrol in HepG2 cells via inhibition of MMP-2 and MMP-9 expressions. IHC confirmed the strong expression of Bax and nuclear expression of PCNA in treated cells. To the best of our knowledge, limited studies have investigated the impact of coumestrol. The study showed that coumestrol exhibited cytotoxic and apoptotic effects against HepG2 cells, accompanied by inhibition of invasion-related gene expression.

## Graphical Abstract

**Figure:**
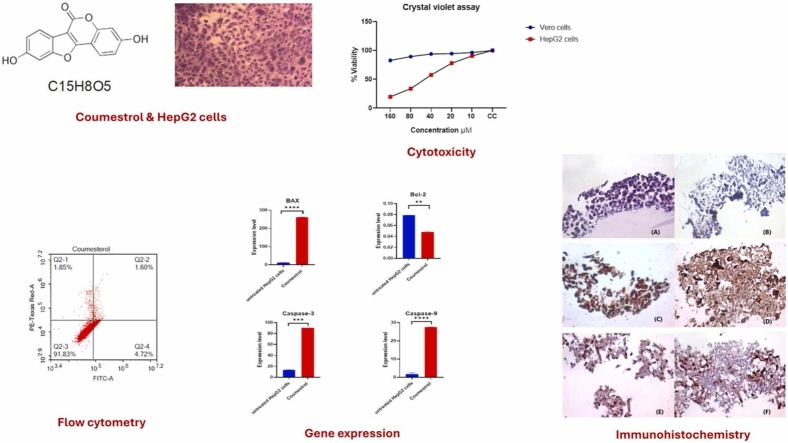

## 1 Introduction

Hepatocellular carcinoma (HCC) is a primary liver tumor that typically occurs in the setting of chronic liver disease/cirrhosis. HCC generally has a poor prognosis, especially among patients with advanced disease [1]. HCC ranks as the sixth most frequently diagnosed cancer worldwide and is the third leading cause of mortality among cancer patients, with approximately 900,000 new cases reported each year. The disease burden is particularly severe in regions with high rates of hepatitis B and C infections, including sub-Saharan Africa and East Asia. Meanwhile, in Western nations, growing cases of metabolic syndrome and non-alcoholic fatty liver disease are driving an upward trend in HCC incidence [2]. HCC is one of the major public health issues in Egypt, wherein it accounts for 13.54 % and 33.63 % of all cancer cases in women and men, respectively [3].

Treatments that kill cancer cells are commonly toxic to normal cells as well. If even a few cancerous cells remain, they can proliferate to produce a revival of the disease, and, unlike the normal cells, they frequently develop resistance to the toxic substance used against them [4].

In recent years, natural products have become an essential part of cancer therapy as alternative medicine, due to their unique structural features, extensive chemical diversity, and less toxicity [5], [6]. Naturally occurring phytoestrogens, or compounds with estrogenic properties found in specific plants, include coumestrol (3,9-Dihydroxy-6-benzofuran [3,2-c] chromenone). It is classified as a dietary isoflavonoid and a member of the coumestan group of compounds [7]. Coumestrol has been found in many plants, including clover, alfalfa sprouts, soybeans, and other legumes. It has acquired popularity because of its possible health advantages and propensity to have estrogenic effects on the body. According to some studies, coumestrol may have anti-inflammatory, antioxidant, and anticancer activities [8].

HepG2 is a widely used human liver cancer cell line. It is widely used in research to investigate toxicity, medication metabolism, and liver problems. HepG2 cells are regarded to be a helpful model for studying the biology of liver cells and several aspects of liver cancer [9].

Anticancer therapies can be devised to selectively eliminate cancer cells by taking advantage of the characteristics that distinguish them from normal cells, such as deficiencies in their DNA repair systems, cell-cycle checkpoints, and apoptosis pathways. The expanding understanding of the biology of cancer in a variety of tumor types and tumor growth is progressively leading to better ways of treating the disease [10]. In HCC, molecular indicators of prognosis include the proliferating cell nuclear antigen (PCNA), and matrix metalloproteinases (MMPs) enzymes have been associated with tumor progression, invasion, and metastasis, which can contribute to a worse prognosis [11]. Moreover, in HCC, alterations in the expression or activity of caspase proteins involved in apoptosis have been reported. Additionally, Bcl-2 is an anti-apoptotic protein that plays a role in regulating cell survival. In HCC, overexpression of Bcl-2 has been associated with a poorer prognosis. Although studies on Bcl-2 in HCC are sparse, some evidence suggests that Bcl-2 downregulation may be related to tumor growth and poor prognosis [12].

Several factors, including research population, specific tumor characteristics, and evaluation methodology, can influence the prognostic significance of these molecular indicators. Their utility as reliable prognostic markers in HCC will need to be established through future research and clinical testing. The abnormal activation of the oncogenic phosphoinositide 3-kinase/protein kinase B/mammalian target of rapamycin (PI3K/AKT/mTOR) signaling is associated with HCC, drives cell survival and proliferation [13]. Coumestrol has shown promise in modulating this pathway and inducing apoptosis via Bax/Bcl-2 and caspase activation, as demonstrated in prior studies [14], [15]. In this work, we aimed to study the cytotoxic effect of naturally occurring coumestrol on HepG2 cells.

## 2 Materials and methods

### 2.1 Cell culture and conditions

The present work involved two cell lines; an African green monkey kidney (Vero cell line), and a human hepatocellular carcinoma (hepG2) cell line, which were kindly obtained from Vacsera, Giza, Egypt. Vero Cell line was cultured and maintained in 25 ml growth medium of Dulbecco’s modified Eagle’s medium with Earle’s salt (DMEM), supplemented with 10 % fetal bovine serum (Biowest) and 100 U/ml each of penicillin/streptomycin and fungizone solution (Lonza). Additionally, a 1 % HEPES buffer (Biowest) was added. While hepG2 cells were maintained in 25 ml RPMI growth medium supplemented with 10 % fetal bovine serum and 100 U/ml each of penicillin/streptomycin and fungizone solution. Afterward, cells were placed in a CO_2_ (5 %) humidified incubator at 37 °C. Ethical approval of the work was gained through the Research Ethics Committee (REC) – Faculty of Science - Ain Shams University, which has approved the current research project. Code: ASU-SCI/ZOOL/2023/3/1.

### 2.2 Crystal violet assay for determining the viability of cultured cells

The antiproliferative efficacy of the coumestrol molecule was estimated through a colorimetric crystal violet (CV) assay. Cytotoxicity of coumestrol was tested against Vero cells (kidney of an African green monkey) and HepG2 (human hepatocellular carcinoma cell line). 1 mg of coumestrol was dissolved in 1 ml of DMSO to get a working concentration of 1 mg/ml. The working concentration was prepared freshly and filtered through a 0.45-micron filter before each assay. Briefly, the two cell lines at a concentration of 1 × 10^5^ cells/well were precultured in 96-well plates for 24 h. Thereafter, based on preliminary dose-response experiments and literature reports, each cell line was separately exposed to varying coumestrol doses, namely control (untrated), 10, 20, 40, 80, and 160 μM for 24 h in a humidified 5 % CO_2_ incubator at 37 °C [8], [16]. Doxorubicin was used as a reference control allowing for the reliable assessment of coumestrol as a cytotoxic agent. After treatment, each cell line was subjected to PBS washing twice followed by CV staining (20 µl) with incubation for 10 min. ELISA plate reader (ELX 800; Bio-Tek Instruments, USA) was used to record absorbance for estimation of optical density (OD) at 490 nm [6].

Treated cells were compared to control cells and the percentage of viable cells at each concentration was calculated. The IC50 (half maximal inhibitory concentration) is a key measure used to determine the effectiveness of a substance in inhibiting a specific biological function, such as cell growth. IC50 was detected for Vero cells and HepG2 cells. By plotting a dose-response curve of the concentration of coumestrol (x-axis) against the percentage of viable cells (y-axis), IC50 or the concentration at which 50 % of the cells were inhibited was calculated [17], [18].

Cell viability (%) = Mean OD/Control OD x 100 % [19]. All experiments were performed in triplicate, and the mean values were used for calculation. Doxorubicin was used as the standard drug [18].

### 2.3 Selectivity index (SI)

The SI indicates the cytotoxic selectivity (i.e. safety), the dosage range in which a compound is effective without serious adverse effects. The SI is the proportion of the IC50 obtained from the experiment on normal cells vs. cancer cells.

SI = IC50 of a compound in a normal cell line/ IC50 of the same compound in a cancer cell line the higher the therapeutic index, the safer the drug. The lower the therapeutic index, the more risk there is for adverse reactions and toxic effects [20].

### 2.4 Annexin V-FITC assay for apoptosis assessment

Apoptotic and necrotic cell populations were determined using Annexin V-FITC apoptosis detection kit (Abcam Inc., Cambridge Science Park, Cambridge, UK) coupled with 2 fluorescent channels flow cytometry. Briefly, HepG2 cells were cultured in a 6-well plate in RPMI media supplemented with 10 % fetal bovine serum (Biowest) and 100 U/ml each of penicillin/streptomycin and fungizone solution (Lonza), in a humidified, 5 % (v/v) atmosphere at 37 °C. Cells were incubated for 12 h, followed by coumestrol treatment at a dose of 60 μM, for 48 h. After treatment with coumestrol for the specified duration, cells 2 x 10^6^ cells) were collected by trypsinization and washed twice with ice-cold PBS (pH 7.4). Then, cells were incubated in the dark with 0.5 ml of Annexin V-FITC/PI solution for 30 min at room temperature according to manufacturer protocol. After staining, cells were injected via ACEA Novocyte™ flow cytometer (ACEA Biosciences Inc., SanDiego, CA, USA) and analyzed for FITC and PI fluorescent signals using FL1 and FL2 signal detector, respectively (λex/em 488/530 nm for FITC and λex/em 535/617 nm for PI). For each sample, 12,000 events were acquired and positive FITC and/or PI cells were quantified by quadrant analysis and calculated using ACEA NovoExpress™ software (ACEA Biosciences Inc., San Diego, CA, USA) [21], [22].

### 2.5 Detection of relative expression of BAX, bcl-2, NF-Kβ, PCNA, MMP2, Cas3, Cas9, COX2, and MMP9 using quantitative real-time PCR (qPCR)

HepG2 cells were cultured for 24 hrs at 37 ℃ in the 12-well plate as explained above with coumestrol (at 60 µM) and without coumestrol. DNA/RNA extraction kit (INtRON Biotechnology) according to manufacturer’s instructions. Synthesis of cDNA was done by using the Thermoscientific RevertAid First Strand cDNA synthesis kit (K1622) according to the manufacturer’s instructions. Thermoscientific Maxima SYBR Green qPCR Master Mix (2x) (K0251) was used to execute the PCR reactions. The relative expression of the target genes (BAX, bcl-2, NF-Kβ, PCNA, MMP2, Cas3, Cas9, COX2, and MMP9) was calculated against the reference gene, GAPDH. The sequences of primer are shown in Table 1. The relative expression was determined using the comparative cycle threshold (Ct) (2^−ΔΔCT^) method [23].

**Table 1:** Sequence of primers used for gene expression analysis using qPCR.

Gene name	Accession number	Primer sequence	Product size
PCNA	BC000491.2	F- 5’-CAAGTAATGTCGATAAAGAGGAGG−3’ R- 5’-GTGTCACCGTTGAAGAGAGTGG−3’	126 bp
NF-Kβ	XM_054369602.1	F−5’-CGCAAAAGGACCTACGAGAC−3’ R- 5’-TGGGGGAAAACTCATCAAAG−3’	193 bp
Caspase−3	XM_054350958.1	F: 5’-ATGGAAGCGAATCAATGGA−3’ R: 5’-TGTACCAGACCGAGATGTC−3’	137 bp
Caspase−9	XR_007064158.1	F−5’-GACGCCATATCTAGTTTGCCC−3’ R- 5’-CACTGCTCAAAGATGTCGTCC−3’	134 bp
MMP−2	BC002576.2	F- 5’ -GGGACAAGAACCAGATCACATAC−3’ R- 5’-GTGGATACGAGAAAACCGCAG−3’	134 bp
MMP−9	NM_004994.3	F- 5’-ATCCAGTTTGGTGTCGCGGAGC−3’ F−5’ -GAAGGGGAAGACGCACAGCT−3’	125 bp
BAX	XM_047439168.1	F- 5’ -GGTTGTCGCCCTTTTCTA−3’ R- 5’ -CGGAGGAAGTCCAATGTC−3’	108 bp
Bcl2	XM_047437733.1	F- 5’-GATGTGATGCCTCTGCGAAG−3’ R- 5’-CATGCTGATGTCTCTGGAATCT−3’	92 bp
COX2	AY462100.1	F- 5′-CGGTGAAACTCTGGCTAGACAG −3′ R- 5′-GCAAACCGTAGATGCTCAGGGA −3′	156 bp
GAPDH	NM_001357943.2	F- 5’ -CATGAGAAGTATGACAACAGCCT −3’ R−5’ -AGTCCTTCCACGATACCAAAGT −3’	113 bp

### 2.6 Histopathological analysis, Immunohistochemistry staining, and interpretation

#### 2.6.1 Histological examination

Preparation of cultured cells fixed in formalin was done according to Koh et al. (2013). The HepG2 cells were grown on culture plates till reaching 80 % confluence. Then, cells were removed by trypsinization. Cells were harvested by centrifugation at 1000–3000 x g for 1 min. Cells were washed twice with 20 ml of 1x PBS (Biowest, USA). After that, centrifugation was performed and cells were resuspended in 20 ml of 10 % neutral buffered formalin (VWR, Cat. #VW3239–4) and were incubated for 15 min at room temperature. Centrifugation and washing steps were repeated. Finally, 0.5 ml of 3 % low melting agar solution (Sigma, USA, Cat Number: 39346–81–1) was added. The agarose pellet was then placed into a tissue cassette for tissue processing. Four microns thick sections were stained with hematoxylin and eosin stain (H and E stain) for histopathological examination [24].

#### 2.6.2 Immunohistochemical analysis of Bax and PCNA expression

Removal of wax from the cells’ sections was done by immersing the slides in xylene with two changes for 10 min each, followed by rehydration of tissue sections by immersing the slides in decreasing grades of ethanol. Antigen retrieval was established using a PT link solution and incubator (DAKO). The slides were washed three times; 3 min each, by immersing them in BPS. Quenching of endogenous peroxidase was done by incubating the slides in 3 % hydrogen peroxide prepared in methanol for 15 min in dark conditions. Then, the slides were washed in TBST 3 times, 3 min each. Each antibody for Bax (anti-Bax anti-body, DAKO, catalog: A3533, polyclonal) and PCNA (Anti-PCNA antibody, DAKO, P04961 - monoclonal) was used at a dilution 1:100 in 1x PBS. Sections were incubated with diluted primary antibody overnight, at 4◦C, in a humidified chamber. After washing the 4 times, 5 min each with TBST, they were then incubated for 30 min with the secondary biotinylated antibody followed by avidin-peroxidase complex (Avidin peroxidase Universal Detection Kit, Dako, Denmark) for another 30 min according to the manufacturer’s instructions. A brown color was developed with diaminobenzidine for 2–4 min, washed in distilled H_2_O, and counterstained with Mayer’shematoxylin for 1 min. The entire procedure was performed at room temperature. In addition, negative controls, in which the primary antibody was omitted and replaced by 1x PBS, were also used. The expression level of Bax and PCNA in tissue cells was judged according to the percentage of Bax and PCNA-positive cells in each sample. Specifically, a percentage of ≤ 10 % was judged negative, and > 10 % was positive. All sections were evaluated and recorded. The sections were examined using a light microscope [Scope A1, Axio, Zeiss, Germay]. Photomicrographs were taken using a microscope camera [AxioCam, MRc5, Zeiss, Germany].

### 2.7 Statistical analysis

All experiments were performed in technical triplicates. All the statistical details of the experiments can be found in figure legends, figures, and results. The results were expressed as mean ± standard error of means (SEM). A comparison between coumestrol untreated and treated HepG2 cells was performed using Student’s *t*-test. GraphPad Prism 8 software was used to generate graphs and conduct all statistical analyses. p-value ≤ 0.05 was considered significant.

## 3 Results

### 3.1 Cytotoxicity effects of coumestrol

**Fig. 1:**
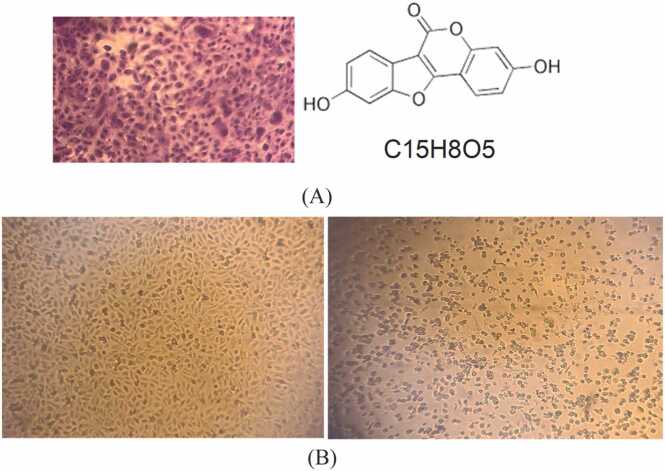
(A) On the right: coumestrol chemical structure, on the left: cell morphology of HepG2 cells examined with an inverted microscope (20x magnification) showing 100 % confluent sheet, viable cells are stained with crystal violet. (B) on the right: cell morphology of HepG2 cells examined with an inverted microscope (20x magnification), HepG2 cells treated with 160 µM coumestrol showing cytotoxicity of 80.3 %, on the left untreated HepG2 cells showing 100 % confluent sheet.

**Fig. 2:**
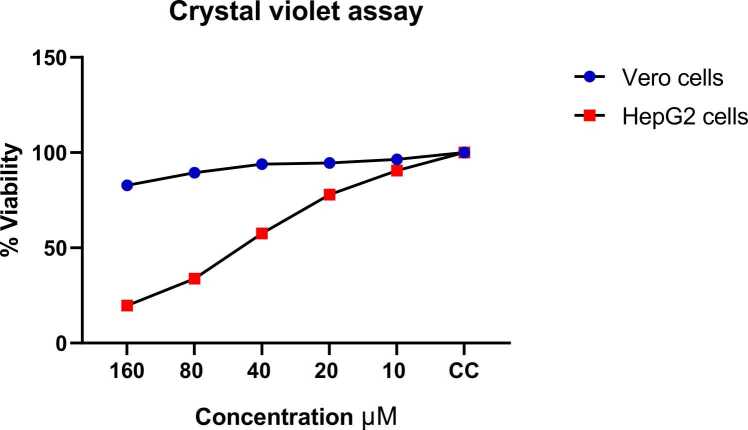
Dose-dependent cytotoxic effect of coumestrol on HepG2 liver cancer cells and Vero normal cells assessed by crystal violet viability assay. Cells were treated with increasing concentrations of coumestrol (10, 20, 40, 80, and 160 μM) for 24 h at 37 °C. Cell viability is expressed as percentage relative to untreated control cells (CC). The graph demonstrates selective cytotoxicity of coumestrol against HepG2 liver cancer cells (red squares), showing a concentration-dependent decrease in viability from approximately 100 % at the lowest concentration to 20 % at 160 μM. In contrast, Vero normal cells (blue diamonds) maintained high viability (80–100 %) across all tested concentrations, indicating minimal cytotoxic effects on non-cancerous cells. Data points represent mean values, and the results suggest that coumestrol exhibits preferential toxicity toward cancer cells while sparing normal cells, demonstrating its potential as a selective anticancer agent. CC = cell control (untreated cells, 100 % viability reference).

**Fig. 3:**
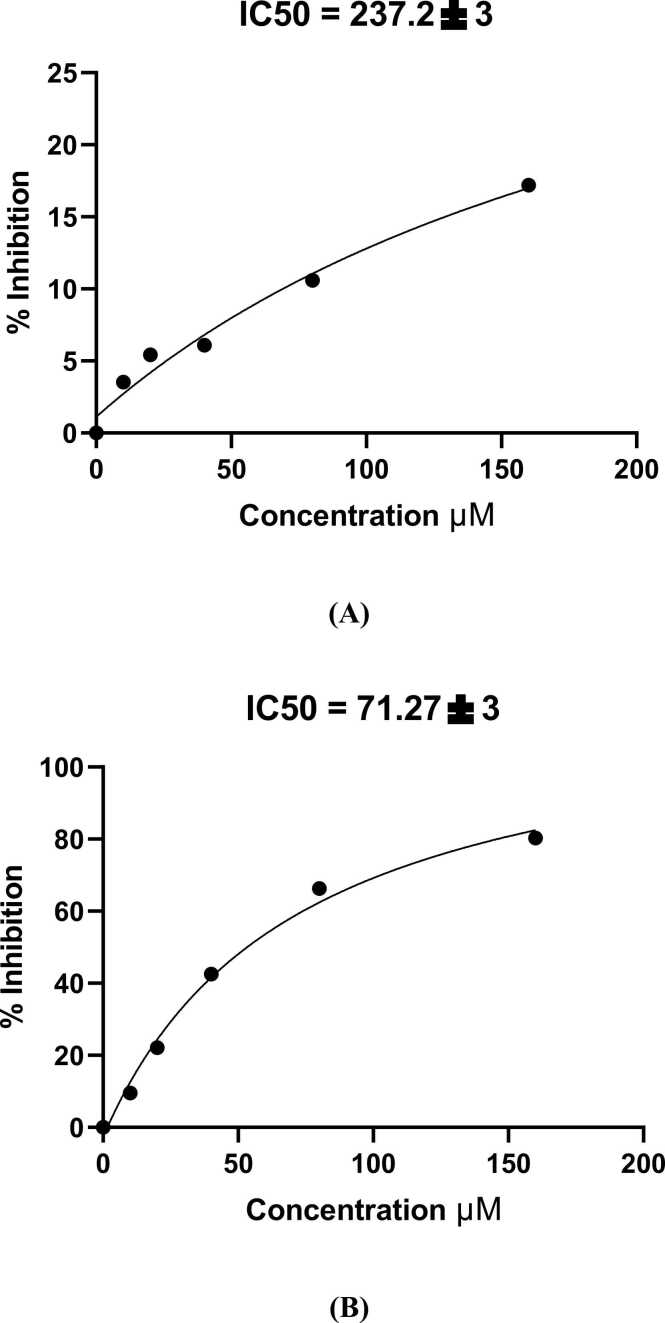
(A): Percentage of inhibition of Vero cells at different concentrations of coumestrol with IC50 value. (B)**:** Percentage of inhibition of HepG2 cells treated with various concentrations of coumestrol with IC50 value.

HepG2 cell proliferation was examined using CV assay. Results represented that the proliferation of these cells decreased in a dose-dependent manner. The 50 % cytotoxic concentration of coumestrol required to reduce cell viability of Vero cells by 50 % was calculated. The IC50 was plotted using a dose-response curve, and the selective index (SI) for coumestrol was estimated by dividing the IC50 on Vero cells with the IC50 on HepG2 cells. Coumestrol didn’t affect the growth of Vero cells, the proliferation rate remained nearly intact. The IC50 of coumestrol was typically 71.27 µM, indicating effective inhibition of HepG2 cell growth. For Vero cells, the IC50 was higher (237.2 µM) indicating that coumestrol is less toxic to normal cells compared to cancer cells. Nevertheless, coumestrol was less active than doxorubicin (IC50 of doxorubicin on HepG2 cells = 22 µM) but was more toxic to HepG2 cells than Vero cells (Table 2 and Fig. 1, Fig. 2, Fig. 3).

**Table 2:** The percentage of cytotoxicity of coumestrol on Vero cells and HepG2 cells using different concentrations of coumestrol.

**Concentration** **μM**	**Cytotoxicity**		
	**Vero**	**HepG2**	
**160**	17.2 %		80.3 %
**80**	10.6 %		66.25 %
**40**	6.1 %		42.5 %
**20**	5.43 %		22.1 %
**10**	3.53 %		9.5 %

### 3.2 Selectivity index (SI)

The SI is a measure of the safety and efficacy of a drug, calculated as the ratio of the toxic dose to the effective dose. According to the IC50 of coumestrol on Vero cells and IC50 on HepG2 cells, the SI was calculated where the IC50 of Vero cells was 237.2 µM and the IC50 of HepG2 cells was 71.27 µM. Accordingly, the SI was IC50 of coumestrol in the Vero cell line/ its IC50 in the HepG2 cell line = 237.2/71.27 = 3.3.

### 3.3 Induction of apoptosis by coumestrol in HepG2 cells: flow cytometry

**Fig. 4:**
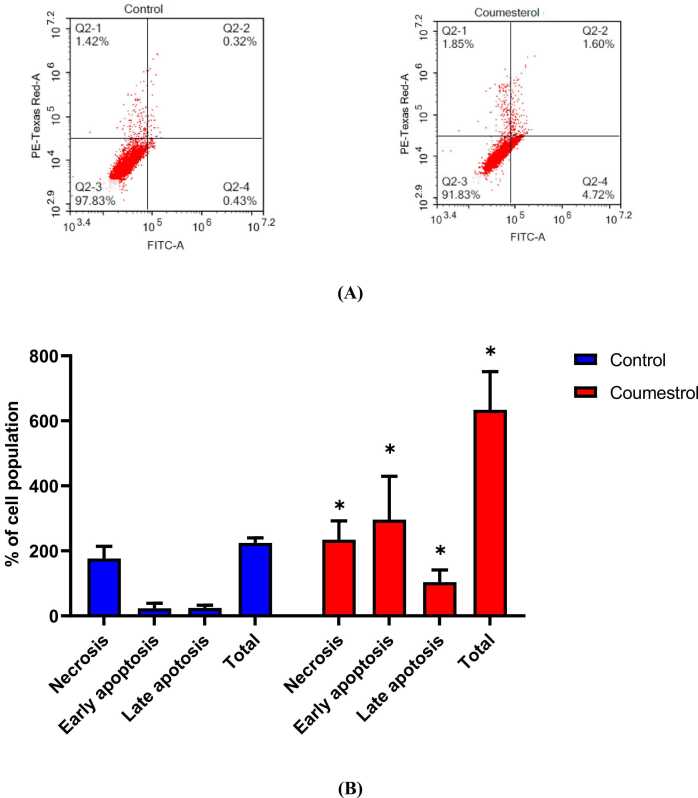
(A) Apoptosis/necrosis assessment in HepG2 cells after treatment with coumestrol for 48 h. Cells were stained with PI/Annexin V-FITC. (B) Different cell populations were plotted as the percentage of total events. Data are presented as mean ± SD; n = 3. * Statistically significant difference from the control (P < 0.05).

The Annexin V-FITC assay was conducted to evaluate the apoptotic effects of coumestrol on HepG2 cells. Cells were treated for 48 h and analyzed using flow cytometry. The results are summarized in Fig. 4, which shows the percentage of cells in each category, for control untreated HepG2 cells: viable cells (Q3) were 97.83 %, early apoptotic cells (Q4) were 0.43 %, late apoptotic cells (Q2) were 0.32 %, and necrotic cells (Q1) were 1.42 %. On the other hand, for coumestrol-treated HepG2 cells, the percentages were: 91.83 %, 4.72 %, 1.60 %, and 1.85 %, respectively. Statistical analysis revealed significant differences (p < 0.05) between treated and control groups. Thus, the treatment significantly increased the percentage of early and late apoptotic cells compared to the control cells, suggesting that coumestrol effectively induces apoptosis in HepG2 cells.

### 3.4 Molecular analysis using qPCR

**Fig. 5:**
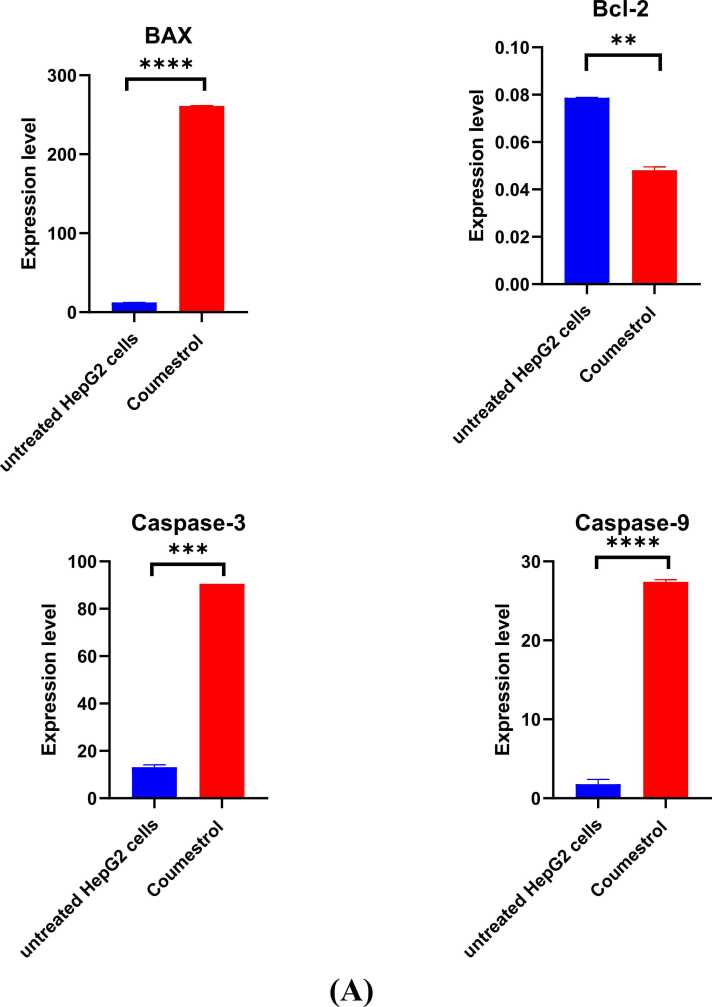

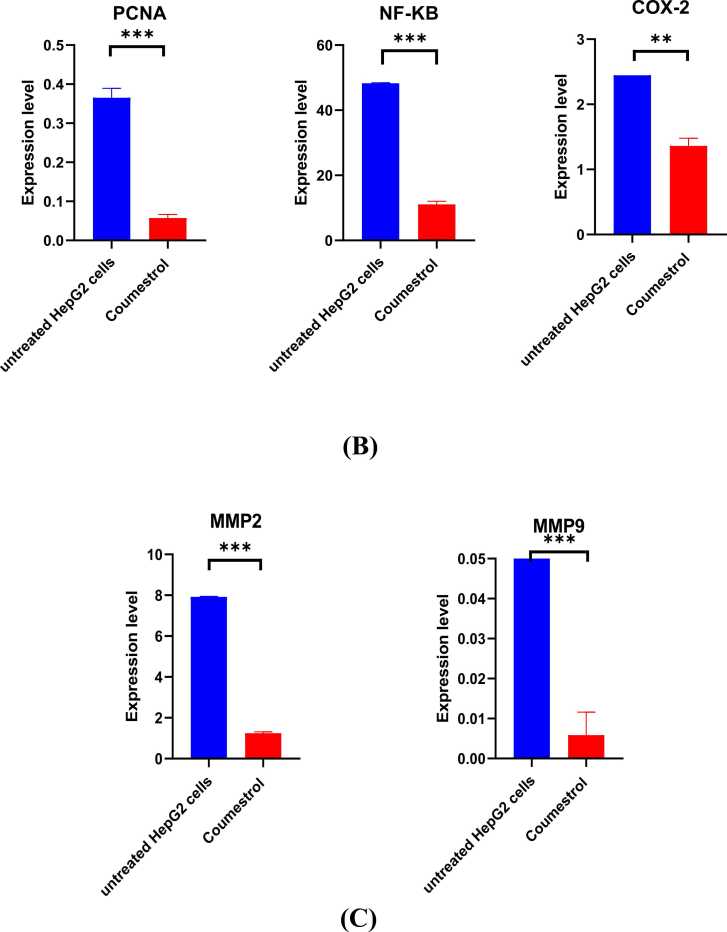
qPCR analysis of mRNA levels in untreated and coumestrol-treated HepG2 cells (conc. 60 µM) for 24 hr. (A) For apoptosis markers; BAX, bcl-2, Cas3, and Cas9. (B) Marker cell of proliferation (PCNA), and Inflammatory response regulators NF-Kβ and COX2. (C) Matrix-modulating proteins MMP-2 and MMP-9. All experiments for each gene were conducted in technical triplicates. ****p < 0.0001 is considered significant.

Molecular analysis of gene expression using qPCR was conducted using the apoptosis markers; BAX, Bcl-2, caspase 3, and caspase 9. The expression of BAX was significantly increased compared to the untreated HepG2 cells potentially leading to apoptosis (*p* = 0.0425). Also, coumestrol treatment caused a decrease in Bcl2 expression (*p* = 0.0341), indicating a potential pro-apoptotic effect of coumestrol on HepG2 cells. Coumestrol increased the expression of both caspase-3 (*p* < 0.0001) and caspase-9 in HepG2 cells (*p* = 0.0030), thus, promoting apoptosis through both intrinsic and extrinsic pathways (Fig. 5. A). PCNA, which serves as a marker of proliferation, was overexpressed in HepG2 cells, and after treatment with coumestrol, its expression was decreased indicating inhibition of cell growth and proliferation in HepG2 cells (*p* < 0.0001)**.** In treated HepG2 cells NF-κB expression was downregulated suggesting that it can promote apoptosis in cancer cells (*p* = 0.0437). COX-2 levels were reduced after treatment, meaning that coumestrol may help lessen inflammation (*p* < 0.0001) (Fig. 5. B. (Treatment with coumestrol downregulated the expression of MMP2 (*p* < 0.0001) and MMP9 in HepG2 cells (*p* = 0.0013) (Fig. 5. C)

### 3.5 Histopathological examination and immunohistochemical analysis

**Fig. 6:**
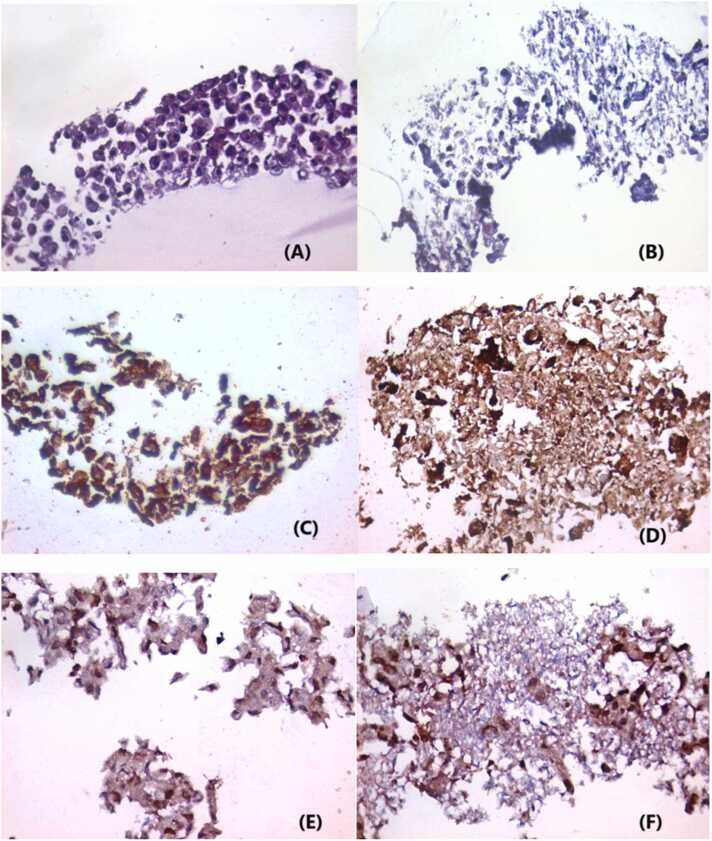
(A) Control cells of intact HepG2 cells with preserved architecture, amphophilic cytoplasm, and hyperchromatic anisochromic nuclei (Hematoxylin and eosin stain, X400). (B) Treated HepG2 cells with 60 µM coumestrol for 24 h, HepG2 cells mostly necrotic with only few scattered preserved cells (Hematoxylin and eosin stain, X400). (C) Control cells of intact HepG2 cells with positive expression of Bax, both nuclear and cytoplasmic (IHC for Bax, using DAB, X400). (D) Treated HepG2 cells with 60 µM coumestrol for 24 h, which shows few degenerated HepG2 cells entangled within tissue necrosis with only strong Bax expression (IHC for Bax, using DAB, X400). (E) Control cells of intact HepG2 cells with positive nuclear expression of PCNA in most cells (IHC for PCNA, using DAB, X400). (F) Treated HepG2 cells with 60 µM coumestrol for 24 h, it shows few degenerated HepG2 cells entangled within tissue necrosis with nuclear expression of PCNA (IHC for PCNA, using DAB, X400).

Histopathological examination of sections prepared from the HepG2 cell control group and stained with H&E showed intact HepG2 cells with preserved architecture, amphophilic cytoplasm, and hyperchromatic anisochromic nuclei. HepG2 cells were mostly necrotic with only a few scattered intact cells after 24 hrs incubation with 60 µM coumestrol) Fig. 6. Studying the expression of PCNA and Bax in tissue sections in control untreated cells and in HepG2 cells treated with 60 µM coumestrol for 24 h showed that control cells were intact HepG2 cells with positive expression of Bax, both nuclear and cytoplasmic. Treated HepG2 cells with 60 µM coumestrol for 24 h, showed only a few degenerated HepG2 cells entangled within tissue necrosis exhibiting strong Bax expression. For PCNA expression, control cells showed intact HepG2 cells with positive nuclear expression of PCNA in most cells, on the other hand, treated HepG2 cells with 60 µM coumestrol for 24 h, showed few degenerated HepG2 cells entangled within tissue necrosis with nuclear expression of PCNA, as shown in Fig. 6. Thus, the results conclude the obvious therapeutic toxic effect on HepG2 cells, compared to control cell line.

## 4 Discussion

The studies conducted on plant-derived chemicals as anticancer medications emphasize their potential as safer alternatives to standard chemotherapy [25]. While research continues to clarify the mechanisms and effects, these natural compounds are an important resource in the ongoing fight against cancer. HepG2 is a popular hepatic cell line. It is used in a wide range of studies, from the oncogenesis to the cytotoxicity of substances in the liver [9]. Here, we investigated the potential effects of coumestrol on HepG2 cells to understand the mechanism involved and to determine the therapeutic implications. The effects of coumestrol, like any other bioactive compound, can vary based on the concentration, duration of exposure, and the individual cellular context [26].

The crystal violet assay is an efficient tool to assess cytotoxicity and cell viability because of its simplicity and reproducibility [27]. In this study, we demonstrated that coumestrol effectively inhibits the proliferation of HepG2 cells in a dose-dependent manner, with an IC50 value of 71.27 µM, indicating significant cytotoxicity towards cancer cells. In contrast, coumestrol exhibited a much higher IC50 of 237.2 µM on Vero cells, suggesting selective toxicity that spares normal cells while targeting cancerous ones. The study of Moustafa et al. (2014), evaluated the cytotoxic activity as a remarkable activity when affecting > 75 % of the cell population, moderate activity when affecting 75–40 % of the cell population, low activity when affecting 40–0.1 % of the cell population, or no cytotoxic activity when affecting 0 % of the cell population [28]. Coumestrol had low cytotoxic activity against normal Vero cells (17.2 % at 160 µM) and had a remarkable cytotoxic effect against HepG2 cells (80.3 % at 160 µM coumestrol). Coumestrol showed selective action against HepG2 cells while maintaining a relatively low toxicity profile for normal cells, this is a desirable trait in anticancer drug development. These results highlight coumestrol's potential as an anticancer agent, although it was less potent than doxorubicin, which had an IC50 of 22 µM against HepG2 cells. These findings align with the study conducted by Kuang et al. who demonstrated that coumestrol induced significant toxicity in human skin cancer cells SKEM-5, while the proliferation rate in normal skin cells remained almost intact [7].

The data obtained in the current study revealed that the selectivity index (SI) for coumestrol on HepG2 cells is 3.3. Senthilraja and Kathiresan (2015) in their study stated that the higher the SI, the safer the drug. An SI of 3.3 suggests that the effective dose is significantly lower than the toxic dose, it indicates that coumestrol has a relatively high margin of safety, with the therapeutic dose being 3.3 times lower than the toxic dose which generally indicates a relatively safe therapeutic window. It implies that there is a substantial difference between the doses that produce therapeutic effects and those that produce toxicity [19].

Apoptosis is a potential strategy to inhibit carcinogenesis by stopping uncontrolled cell growth. Apoptosis executes as a defensive mechanism that eliminates impaired or harmful cells preceding the appearance of malignancy. Hence, we conducted flow cytometry analysis using the Annexin V-FITC assay which demonstrated that coumestrol induces apoptosis in HepG2 cells, underscoring its potential therapeutic applications in cancer treatment as clarified by another study [7].

The genes BAX, Bcl-2, NF-kB, PCNA, MMP2, Cas3, Cas9, COX2, and MMP9 are appropriate for investigating HepG2 cells treated with anticancer medications. These genes have crucial roles in cellular processes such apoptosis (BAX, Bcl-2), inflammation (NF-kB), proliferation (PCNA), and metastasis (MMPs). qPCR can effectively assess their expression levels, providing insights into the molecular underpinnings of the treatment of cancer [29], [30], [31].

The expression of BAX significantly increased compared to the untreated HepG2 cells, this suggests that coumestrol treatment induced the upregulation of BAX, potentially leading to apoptosis. Also, coumestrol treatment caused a decrease in Bcl2 expression, indicating a potential pro-apoptotic effect of coumestrol on HepG2 cells. NF-κB is often associated with cell survival and proliferation, in treated HepG2 cells its expression was downregulated suggesting that it can promote apoptosis in cancer cells. PCNA serves as a marker of cellular proliferation [32], it was overexpressed in HepG2 cells and after treatment with coumestrol, its expression decreased indicating inhibition of cell growth and proliferation in HepG2 cells. Moreover, MMP-2, a collagenase IV is involved in tissue remodeling and tumor invasion by degrading extracellular matrix components, MMP-9 is involved in developing malignancies and enhances tumor angiogenesis, progression, invasion, and metastasis [33]. Treatment with coumestrol downregulated the expression of MMP2 and MMP9 in HepG2 cells. Coumestrol increased the expression of both caspase-3 and caspase-9 in HepG2 cells, thus promoting apoptosis through both intrinsic and extrinsic pathways. Finally, Cyclooxygenase-2 (COX-2) is an important mediator of angiogenesis and tumor growth [34], COX-2 levels were reduced after treatment, meaning that coumestrol may help lessen inflammation.

The observed upregulation of pro-apoptotic markers (BAX, caspase-3, caspase-9) and downregulation of anti-apoptotic and proliferative markers (Bcl-2, PCNA) align with the established mechanisms of coumestrol action. The suppression of matrix metalloproteinases (MMP2, MMP9) further suggests an anti-invasive potential, which is crucial given the aggressive and metastatic nature of HCC. Recent studies have emphasized the importance of targeting MMPs to inhibit tumor invasion and metastasis, supporting the relevance of our findings [35].

Immunohistochemistry (IHC) is an important tool for supporting qPCR results, especially in cancer diagnoses and research. IHC gives visual confirmation of protein expression in tissue samples, allowing for a link between the presence of specific proteins and gene expression data obtained using qPCR. The combination of IHC and qPCR can improve prognostic evaluations [36], [37]. Here, HepG2-treated cells showed disordered cells with extensive necrosis. Bax expression was mostly cytoplasmic, the level of Bax expression was enhanced with coumestrol treatment, suggesting that coumestrol promotes pro-apoptotic signals. PCNA expression in coumestrol-treated HepG2 cells was mostly nuclear. The decrease in PCNA expression following coumestrol treatment indicates that coumestrol inhibits cell growth and proliferation in HepG2 cells, which is consistent with its role as an anticancer agent.

## 5 Conclusion

The study demonstrated that coumestrol possessed cytotoxic capabilities against HepG2 cells, a model for liver cancer. Coumestrol-induced apoptosis by increasing pro-apoptotic signals (Bax and caspase-3 activity) while potentially suppressing anti-apoptotic signals (Bcl-2). It lowered PCNA levels and may alter inflammatory pathways (NF-κB and COX-2) and matrix remodeling processes (MMPs) leading to apoptosis of HepG2 cells. It stopped the cell cycle, halting the continued division and development of HepG2 cells. Coumestrol successfully inhibited the growth of HepG2 cells. Given the limited treatment options and poor prognosis associated with advanced HCC, the ability of coumestrol to target key pathways involved in cancer cell survival and proliferation is particularly significant. Nevertheless, further preclinical *in vivo* studies are necessary to assess its biological activity and toxicity to fully explore coumestrol's therapeutic potential and to optimize its use in the treatment of HCC. Further studies would be required to elucidate the precise molecular mechanisms involved. While this study focuses on HepG2 cells, future research could explore the effects of coumestrol on other cancer types to broaden its therapeutic applications.

## Limitations and future perspectives

We acknowledge the limitation of using a non-human Vero cell line which is derived from African green monkey kidney tissue, which may not represent normal human hepatocytes. However, we opted for Vero cells due to their established role in cytotoxicity assays and their ability to provide preliminary insights into cellular responses. Vero cells have been widely utilized in various cytotoxicity studies, including those involving compounds with potential therapeutic applications, due to their robust growth and ease of handling. Moreover, Results are based solely on the HepG2 cell line, which may not represent the diversity of HCC. Further studies using other human hepatocyte cell lines are warranted to validate our results.

Moreover, another limitation in our study is the use of the crystal violet (CV) assay. The assay is a well-established, peer-reviewed method for cytotoxicity assessment in oncological research. Its reliability is demonstrated by its widespread validation in literature for adherent cell lines like HepG2. It provides direct correlation with cell density through DNA staining, avoiding metabolic confounders. While the CV assay reliably measures cell viability based on total cell biomass, it does not provide information on specific metabolic activity or distinguish between different forms of cell death, which could be addressed by complementary assays such as XTT, MTT, or CellTiter-Glo.

Also, a limitation of our study is that protein-level confirmation of all qPCR results by Western blot, as well as the inclusion of additional immunohistochemical markers, was not feasible within the current scope. Future studies should address these aspects to provide a more comprehensive validation of molecular changes.

Furthermore, the findings are based on *in vitro* experiments, which may not accurately reflect *in vivo* conditions and interactions. So, we aim to adopt *in vivo* studies on animals in the future to justify the results. Finally, our research focused on a narrow range of molecular markers, potentially overlooking other important mechanisms involved in coumestrol's effects is important for further exploration of underlined mechanisms. Continued discovery of the molecular pathways involved in HCC progression will likely uncover new therapeutic targets and improve understanding of disease mechanisms and to fully understand coumestrol's therapeutic potential in HCC treatment.

## CRediT authorship contribution statement

**Nagui Fares:** Writing – review & editing, Validation, Supervision, Methodology, Conceptualization. **Faten Sabra Abo-Zeid:** Writing – review & editing, Validation, Supervision, Methodology, Conceptualization. **Hoda Abdul-Nabie:** Writing – original draft, Validation, Methodology, Investigation, Formal analysis, Conceptualization. **Tarek Aboshousha:** Writing – original draft, Validation, Methodology, Investigation, Formal analysis. **Safia Samir:** Writing – review & editing, Writing – original draft, Validation, Supervision, Methodology, Formal analysis, Data curation, Conceptualization.

## Ethics approval

The protocol of the study was approved by the scientific research ethics committee of Ain Shams University / Faculty of Science / Zoology Department, Code ASU-SCI/ Zool / 2023 /3/ 1.

## Funding

Not applicable.

## Declaration of Competing Interest

The authors declare that they have no known competing financial interests or personal relationships that could have appeared to influence the work reported in this paper.

## Data Availability

Data will be made available on request.
